# A Heuristic Machine Learning-Based Optimization Technique to Predict Lung Cancer Patient Survival

**DOI:** 10.1155/2023/4506488

**Published:** 2023-02-02

**Authors:** Sonia Kukreja, Munish Sabharwal, Mohd Asif Shah, D. S. Gill

**Affiliations:** ^1^School of Computing Science and Engineering, Galgotias University, Greater Noida, India; ^2^Kebri Dehar University, Kebri Dahar, Ethiopia; ^3^School of Electrical Engineering and Computer Science, Gwangju Institute of Science and Technology, Gwangju, Republic of Korea

## Abstract

Cancer has been a significant threat to human health and well-being, posing the biggest obstacle in the history of human sickness. The high death rate in cancer patients is primarily due to the complexity of the disease and the wide range of clinical outcomes. Increasing the accuracy of the prediction is equally crucial as predicting the survival rate of cancer patients, which has become a key issue of cancer research. Many models have been suggested at the moment. However, most of them simply use single genetic data or clinical data to construct prediction models for cancer survival. There is a lot of emphasis in present survival studies on determining whether or not a patient will survive five years. The personal issue of how long a lung cancer patient will survive remains unanswered. The proposed technique Naive Bayes and SSA is estimating the overall survival time with lung cancer. Two machine learning challenges are derived from a single customized query. To begin with, determining whether a patient will survive for more than five years is a simple binary question. The second step is to develop a five-year survival model using regression analysis. When asked to forecast how long a lung cancer patient would survive within five years, the mean absolute error (MAE) of this technique's predictions is accurate within a month. Several biomarker genes have been associated with lung cancers. The accuracy, recall, and precision achieved from this algorithm are 98.78%, 98.4%, and 98.6%, respectively.

## 1. Introduction

Due to a close relationship between tumor formation and altered nuclei morphology, nuclear changes have been crucial for cancer diagnosis [1]. Light microscopy (e.g., haematoxylin and eosin) may be used to visually analyze nuclear morphology in clinical diagnosis [2]. In many tumors, pathologists can identify specific nucleus alterations that may be used to guide their treatment options. Numerous numerical parameters [3] that define intrinsic morphological qualities of nuclei, such as their size and shape (e.g., perimeter, area, curvature, and symmetry), as well as nuclear texture, are used in computer-aided diagnostic (CAD) systems to quantify the structure of nuclei [4]. Most of the time, diagnostic labels are only provided for tissue samples, not individual nuclei. A predictive model is required for the set detection issue in order to learn from sets of nuclei without nuclei-level annotations and to anticipate the diagnostic label for a fresh set of nuclei. When a model has to forecast a patient's chance of survival based on a set of measurable nuclei, it is known as the “set detection problem” in cancer diagnosis [5]. Training and testing samples in the set detection scenario are sets, each of which comprises a distinct number of unlabeled nucleus images, while in classic image detection, training and testing samples are labeled single-shot photographs. There is no viable supervised machine learning solution for solving the set detection issue. These nucleus set detection systems and their drawbacks are samples of what is now available in the market. Although it is common for predictive models to make explicit assumptions, this is often done implicitly. A common method for predicting sets is to employ the majority voting strategy, which assumes that at least half of the instances in a collection reflect the category to which the set belongs [6]. Voting thresholds were used to grade hepatocellular cancer tumors in [7]. If want to get the best results, voting thresholds for each class need to be predefined on the basis of on experience in each topic. To be regarded positively, a set must include at least one instance of the positive; otherwise, it is considered negative in the MIL framework [8]. There has been an increasing use of MIL in medical diagnostics [9]. Because of tumor heterogeneity [10], it is often necessary to have prior knowledge of the subject matter to create an accurate prediction model. The prediction model is learned at the set level by using set detection, which takes into consideration the full set of data. Individual nuclei can still be classified, but groupings of nuclei cannot. The most popular and straightforward way [11, 12] is to combine many statistics (STATS) on nuclear feature features into a single set. There are several statistics included in the feature vector that describe the qualities of the nucleus set. Because of this, the effectiveness of STATS is strongly dependent on the experimental data's predesigned statistics. With the help of bag-of-words (BoWs), a method often used in the field of set detection may learn the composition of one set while taking into account the vocabulary included within the training set's collection of sample instances or dictionaries [13].

**Figure 1:**
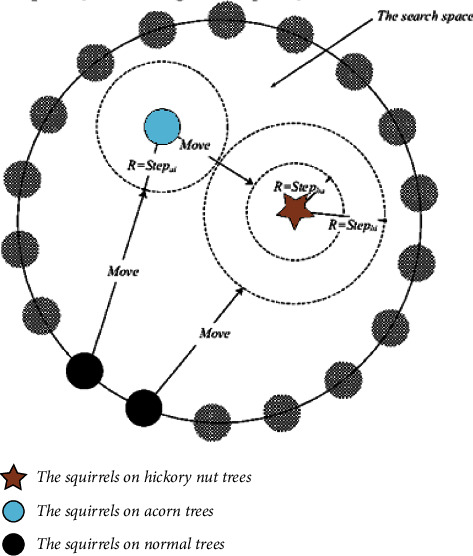
Scenario-based diagram of SSA [14].

Figure 1 depicts the working of the squirrel search algorithm which explains how the squirrels are moving from normal trees to hickory as well as on acorn trees in the search of food. In SSA, each squirrel moves from one position to another position which is a better position. Among all the 3 trees, the hickory tree is considered to be the best tree for food.

The main objectives of this paper are as follows:

1. Provide an efficient Feature selection technique using biomarker genes to find out whether a cancer patient will survive or not.
2. Establish a new method with SSA. If a patient will survive, then the duration is more than five years or not.
3. Design an effective technique to predict the overall survival time with lung cancer.

The structure of this document is as follows.  Section 1 illustrates the introductory part of SSA and the various optimization techniques, and Section 2 outlines some related and motivational work to develop the proposed method.  Section 3 gives a detailed description of the proposed technique, Section 4 depicts the derived results of the proposed technique, and Section 5 provides the conclusion of the proposed work.

## 2. Literature Review

DNA methylation, a critical biomarker in cancer diagnosis, has attracted considerable attention from researchers, who have used a selection of features on the data generated by this biomarker to improve prediction accuracy [15]. In the work of [16], researchers utilized a feature selection strategy. Based on the features of clinical DNA methylation data, a three-step feature selection approach was utilized to identify different cancer- and lymph node-related gene biomarkers. The outcome of this approach reveals a remarkable improvement in the accuracy of prediction in recognizing LN metastasis. The suggested technique employing the K-Nearest Neighbors classification beat previous algorithms on all criteria, and it was able to reliably forecast the expression of individual genes using just DNA methylation data. In addition to being overrepresented in gene ontology concepts related to the control of several biological processes, these DNA methylation-sensitive genes were also shown to be highly expressed. For example, the study of [17] shows the usefulness of feature selection in predicting a wide range of ailments such as lung cancer, heart disease, and so on. It was observed that SVM-RFE, when using support vector machines, had the highest accuracy of 97 percent when comparing the accuracy and efficiency of various feature selection techniques. An additional benefit of using the feature selection strategy to improve classifier accuracy was proven in [18]. Each feature selection approach was shown to act differently and have a unique set of advantages and disadvantages. Random Forest's machine learning method was combined with the feature selection elimination approach in [19]. Researchers set out to create a computer-aided diagnostic system that could distinguish between benign and malignant lung tumors, the first stage of which would undertake data reduction to prepare for the second stage's algorithmic training. A classification accuracy of 99.82% and a precision of 99.70% were achieved using the method proposed in this research. The recent study, on the other hand, has concentrated only on feature extraction methodologies to speed up and improve prediction precision. Colorectal cancer sickness may be predicted using gene expression data, as shown in [20], who suggested a feature extraction technique termed OMBRFE. Singular value decomposition (SVD) was used in this paper's feature extraction approach to reduce the data's high dimensionality. For advanced colorectal cancer in clinical stages, the retrieved genes were revealed to be tightly associated with OMBRFE. To accurately forecast illness, [21] devised a unique feature extraction approach called iterative Pearson's correlation coefficient. (iPcc). In this study, Pearson's correlation coefficient was repeatedly applied to gene expression patterns to build a new set of characteristics for samples [22]. Despite the enormous number of features and the length of time it took to get them, the number of extracted features was equal to the number of samples [23].

The following gaps were identified during the literature review and incorporated into this paper:

- The current work offers a fundamental SSA framework for low-dimension optimization issues that can be expanded to large-scale optimization and constrained optimization situation [24].
- In addition, multiobjective optimization issues may be solved using SSA. The suggested approach may also be used to resolve NP-hard real-world combinatorial optimization issues [25].

## 3. Proposed Algorithm

### 3.1. Squirrel Search Algorithm

The quest starts when flying squirrels begin to forage. When it is warm outside, squirrels glide (fall). They move about a lot, taking in the varied aspects of the forest as they go. It is easier for them to meet their daily energy needs by eating acorns, which are readily available due to the hot climate in the area, and they do so very immediately after discovering them. Once they have consumed their daily caloric needs (hickory nuts), they begin searching for the greatest food source for the winter [26]. Foraging in bad weather is expensive, and hickory nuts will help them satisfy their energy demands, thereby decreasing the need for costly foraging trips. In deciduous woods, a decrease in winter leaf cover raises the risk of predation [27]. After the winter hibernation period is through, the flying squirrels begin to move about again. As a flying squirrel ages, this process continues indefinitely and is the foundation of SSA [28]. When the mathematical model is simplified, the following hypotheses are taken into account:

1. For any deciduous forest, the flying squirrel can be counted on one to perch on the same tree for the whole year.
2. Foraging behaviour of flying squirrels is dynamic, with each squirrel using the resources available to them in the most efficient way possible [29].
3. Only three kinds of trees grow in the forest: hickory trees, normal trees, and oak trees.
4. The *n* in this investigation is set at 50 squirrels. Nutrient food resources (Nfs) are analyzed for four trees [30], one for each of the 46 in the study area: one for the hickory nut tree, and three for the acorn tree. That is, 92% of squirrels are found on trees, with the remainder reliant on food sources for their survival. One ideal winter food supply, however, may be used as a guide for the number of food resources available, where *Z* > 0 is the Nfs number [31].
5. A vector identifies the position of a flying squirrel in a d-dimensional search space. With the ability to change their location vectors, flying squirrels can glide across one-dimensional and two-dimensional search space. The following diagram depicts the SSA process.

### 3.2. Dataset

There are over 100 cases in the Wisconsin Prognostic Lung Cancer subdirectory, which was utilized to build the dataset for this article. The radial distance, opacity, distance from the ground, location, and simplicity of use are some of the characteristics of cancer cell nuclei (local variation in radius lengths). Convexity, rounded edges, and synchronization are all used to gauge how compact something is. Average, standard error, and “worst” are calculated. Data from one lung cancer patient are contained in each entry.

### 3.3. Algorithm Descriptions for Classification Algorithms

Researchers utilized the lung *cancer* dataset to examine the accuracy of three well-known classification methods for the prediction model: Naive Bayes, rapid decision tree learner, and K-nearest neighbor [33]. The next section gives the detailed description of algorithms implemented in this article.

#### 3.3.1. A Naive Bayes Algorithm

The Bayesian classification technique encompasses both supervised learning and statistics categorization. Using probabilities as a basis, one may measure the model's uncertainty using probabilities. It can recognize and anticipate problems [34]. The Bayes theorem is named after this categorization, according to Thomas Bayes (1702–1761). Bayesian classification provides a set of practical learning algorithms that use prior knowledge and observed data [35]. This approach may be used to examine a wide range of learning algorithms. Probability calculations, as well as noise in the data supplied into it, are all handled by this model.

#### 3.3.2. Quick Decision-Making Algorithm for Tree Learners

Regression tree logic is used in iterations of REPTree to generate a large number of trees. Finally, it selects the best-looking tree out of all the trees that were constructed. The tree is also pruned using a backfitting technique in this approach [36]. The values of numerical characteristics are sorted as part of the model preparation process. It is comparable to the C4.5 Algorithm in the way that missing values are handled.

#### 3.3.3. K-Nearest Neighbors Algorithm

According to KNN categorization, the point's nearest neighbors are picked based on how similar they are to each other. An unlabeled example is compared to the other (labeled) examples and the K-nearest neighbors and their labels are calculated to ascertain the sample's classification [37]. Otherwise, it is categorized by either a weighted majority, which gives more weight to points closest to the undescribed object, or by the class that has the majority of the vote for the region.

### 3.4. Algorithms for Selecting Features

For classification, the dataset must be thoroughly examined before being fed into a classifier. When categorizing, it is best to focus on the most important qualities rather than a huge number of insignificant ones. To find the most significant and relevant traits, a broad variety of techniques is necessary. If utilize feature selection to find the most significant features and decrease the load, classification accuracy also rises. In terms of classification accuracy, SSA beats out the competition currently used for feature selection.

Considering the population is N and the upper bound in the search space is represented by FSu, whereas the lower bound has been represented by FS_l._ FSi depicts the population and *i* ranges from 1 to N. D represents dimensions and rand represents a random number. Population is initialized with the help of the following equation:

(1) $$\begin{array}{c} {FS}_{i}={FS}_{l}+rand\left(1,D\right)*\left({FS}_{u}-{FS}_{l}\right)\text{.} \end{array}$$

Equations (2), (3), and (4) are used to identify the position of the squirrel, whether it is on the hickory tree, oak tree, or regular tree, and it can be carried out with the help of

(2) $$\begin{array}{c} {FS}_{at}^{t+1}={FS}_{at}^{t}+dg\times Gc\times \left({FS}_{ht}^{t}-{FS}_{at}^{t}\right),if\;R_{1}>P_{dp}\text{,} \end{array}$$

(3) $$\begin{array}{c} {FS}_{nt}^{t}={FS}_{nt}^{t}+dg\times Gc\times \left({FS}_{at}^{t}-{FS}_{nt}^{t}\right),if\;R_{2}>P_{dp}\text{,} \end{array}$$

(4) $$\begin{array}{c} {FS}_{nt}^{t+1}={FS}_{nt}^{t}+dg\times Gc\times \left({FS}_{ht}^{t}-{FS}_{nt}^{t}\right),if\;R_{3}>P_{dp}\text{.} \end{array}$$

Here, *R* is a random variable that lies between 0 and 1, whereas P_dp_ depicts predator probability of appearance. If *r* > *P*_*dp*_, it means the predator will not appear and vice versa, *t* depicts the current cycle, and *G*_c_ is 1.9. *FSat* represents floating squirrels on an acorn tree, *FSnt* represents floating squirrels on a normal tree, and *FS*_*ht*_ represents floating squirrels on the hickory tree.

In equation (5), *d*_g_ is the floating space that can be calculated with the help of

(5) $$\begin{array}{c} d_{g}=\frac{h_{g}}{\tan\left(\varphi \right)*sf}\text{.} \end{array}$$

In (6), *h*_g_ and sf depict constant values which are 8 and 18, respectively. Now, tan(*φ*) which is the gliding angle will be calculated as

(6) $$\begin{array}{c} \tan\left(\varphi \right)=\frac{D}{L}\text{,} \end{array}$$

where *D* is the pull strength and *L* is the lift strength.

Equation (7) is used to calculate seasonal constant *S*_c_, where *t* = 1, 2, 3.

(7) $$\begin{array}{c} S_{c}^{t}=\sqrt{\overset{d}{\underset{k=1}{\sum }}{\left({FS}_{at,k}^{t}-{FS}_{ht,k}\right)}^{2}}\text{.} \end{array}$$

Some of the advantages of selecting features with SSA include the following: to discover the greatest potential solution, various candidate solutions might explore different sections of the solution space. SSA's solution is an outstanding feature selection tool because it has a memory and can keep knowledge about the solution as it moves across the issue space. Because of its computationally low-cost implementation and good performance, SSA has become a popular choice for many businesses.

As opposed to concentrating on a single response, the Social Security Administration evaluates a broad variety of possibilities. SSA is capable of working with both discrete and binary data. SSA is more efficient in terms of memory and performance than other feature selection approaches. SSA is easy to use, and the results are promising. The scale of the issue has no bearing on SSA's efficacy.

## 4. Experimental Results

The dataset is randomly split into three sets: a training set, a validation set, and a test set in the proportion 7 : 1 : 2. Experiments on each dataset were conducted five times to ensure the fairness and robustness of the proposed technique.

**Figure 2:**
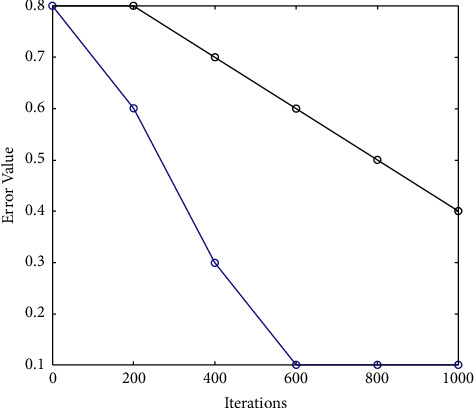
Error rate comparison.

**Figure 3:**
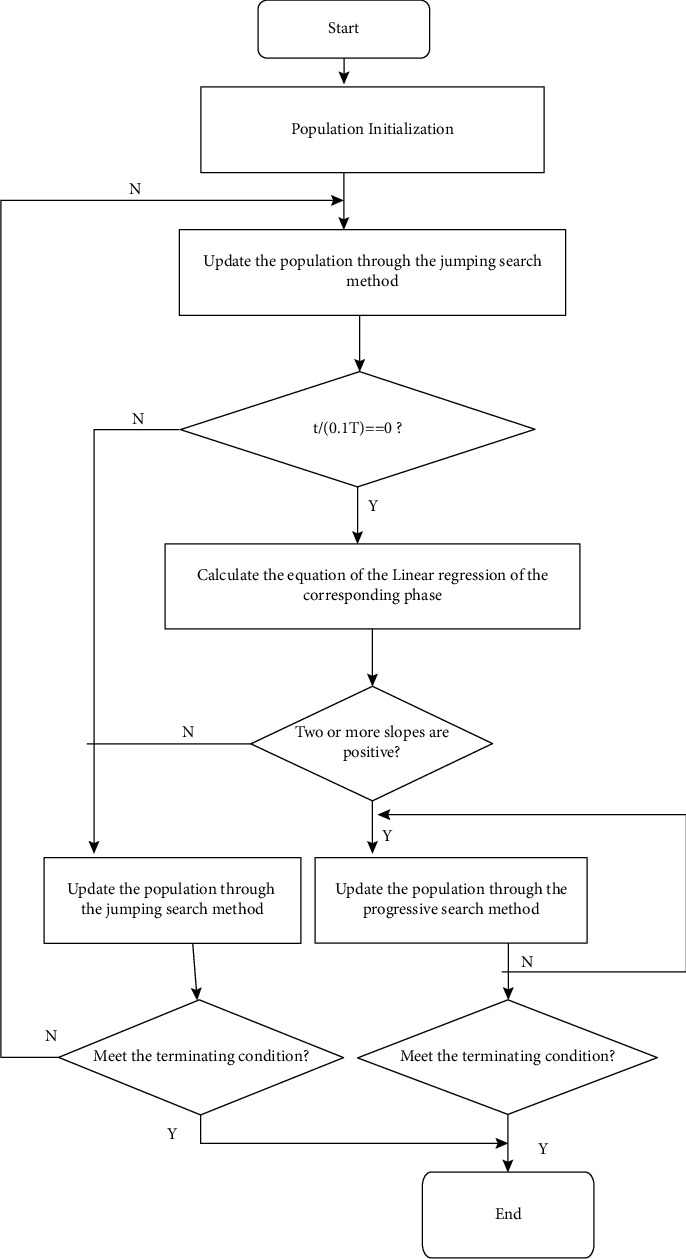
SSA implementation method [32].

Figure 2 illustrates the error value against the iterations. As the iterations increase, an error value is decreasing. In this method, 5 iterations have been conducted on average to achieve the final performance results. The number of iterations has been taken as an input on the *X*-axis from 0–1000, while the error value has been taken on the *Y*-axis. As illustrated in Figure 3, the proposed hybrid approach attained 0.3 less error rate than other existing methods.

Table 1 describes the error percentage value of the proposed work in comparison to the existing algorithm. As illustrated error value has been calculated versus iterations. It is seen that the error rate decreases with the increasing number of iterations. This is due to the optimization of SSA.

**Figure 4:**
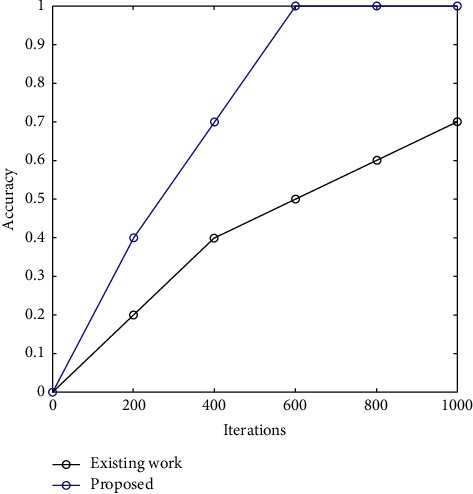
Accuracy comparison of the proposed work.

It is shown in Figure 4 that the suggested approach is more accurate than the current method. Increasing the number of iterations leads to an improvement in accuracy. A large part of this may be attributed to SSA's improved performance. Comparing the suggested method to the current one, it is better at each stage and achieved better accuracy by 5.9% in comparison with the existing method.

Table 2 describes the accuracy rate of the proposed work in comparison to the existing algorithm. As shown, accuracy has been computed with iterations. It is seen that accuracy increased with an increasing number of iterations. This is due to the optimization of SSA.

**Figure 5:**
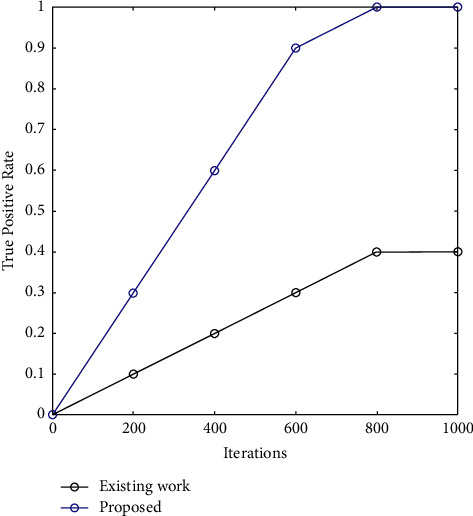
True positive rate of prediction.

A true positive rate comparison of the proposed work is shown in Figure 5. It is evident that the true positive rate shows a gradual increase with the number of rounds. It shows a sudden rise at 600 rounds. The proposed approach has a better true positive rate of 0.6% in comparison to the past approach.

Table 3 describes the true positive rate of the proposed work in comparison to the existing algorithm. True positive has been calculated for iterations, as demonstrated. It is seen that the true positive rate increased by 0.6% with an increasing number of iterations. This is due to the optimization of SSA.

It is possible to obtain rapid convergence in the fusion of two cancers' similarity networks; however, 1,500 iterations are necessary to reach the iteration termination condition. The accuracy and recall of a prediction model are critical metrics for evaluating its performance. Figure 5 depicts the accuracy of the proposed technique. It is clear that as the number of iterations increases, so does the precision which is increased by 10.4%. However, the suggested technique outperforms the current strategy in terms of accuracy and recall. This is due to the application of SSA. The precision rate shows a gradual increase with the number of rounds.

**Figure 6:**
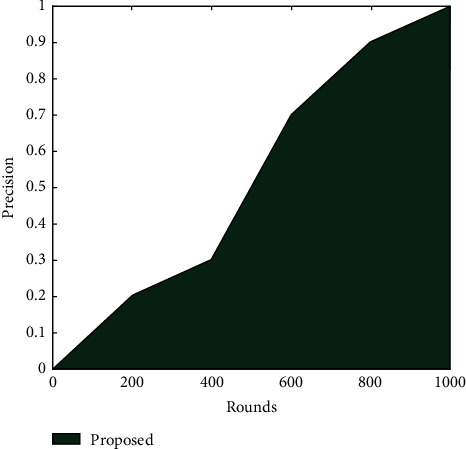
The precision value of the proposed approach.

Figure 6 depicts the precision value of SSA, with the increase in the rounds precision also increases and giving the more accurate result.

Table 4 describes the precision rate of the proposed work in comparison to the existing algorithm. As shown, precision rate has been computed concerning iterations. It is seen that precision rate increased with an increasing number of iterations. This is due to the optimization of SSA.

**Figure 7:**
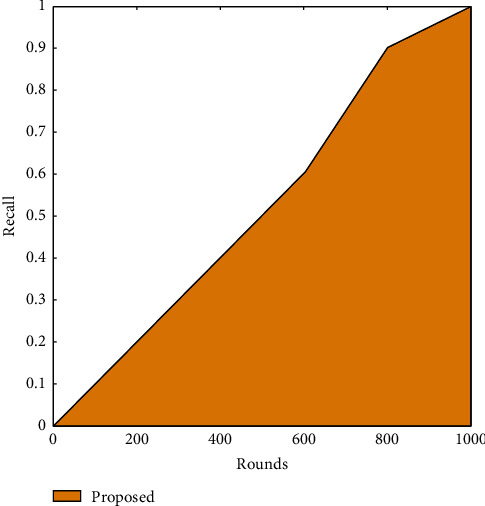
Recall of the proposed algorithm.

It is shown in Figure 7 that increasing the number of iterations leads to an improvement in recall. A large part of this may be attributed to SSA's improved performance. Comparing the suggested method to the current one, it is better at each stage and achieved better recall by 5% in comparison with the existing method.

Table 5 describes the recall value of the proposed work in comparison to the existing algorithm. As shown, the recall value has been computed concerning iterations. It is seen that the recall value increased with the increasing number of iterations. This is due to the optimization of SSA.

And, with this accuracy, precision and recall have been calculated which directly states that this hybrid approach gives better results in comparison to random forest because feature extraction plays an important role in the execution of any technique.

## 5. Conclusion and Future Work

As a part of the investigation into lung cancer prognosis, integrated a feature selection method with a classification system. Using feature selection approaches to minimize the number of features, it is believed that most classification systems may be improved. Certain factors have a greater impact on the categorization algorithms than others. The findings of tests using a well-known classification technique, namely, Naive Bayes+SSA, have been provided. As a result, Naïve Bayes provided superior output without SSA, but SSA enhanced performance in terms of accuracy, precision, and recall, and values obtained are 98.78%, 98.6, and 98.4 in comparison to the random forest which were 92.8, 88.2, and 93.4, respectively. New algorithms and feature selection strategies will be tested in the future as part of this research. These experiments will include both cluster and ensemble methods.

## Figures and Tables

**Algorithm 1:**
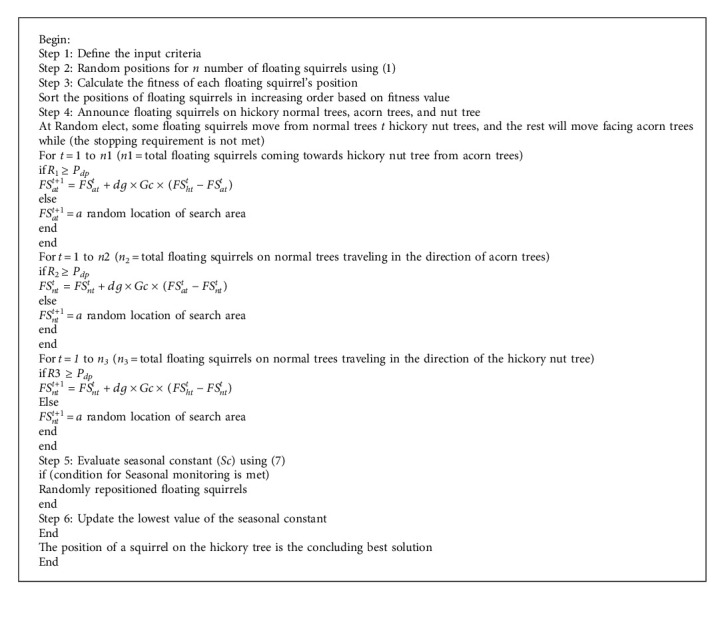
: Squirrel search algorithm.

**Table 1:** Error comparison rate.

Error comparison		
Rounds	Random forest	SSR
0	0.8	0.6
200	0.8	0.3
400	0.7	0.1
600	0.6	0.1
800	0.5	0.1
1000	0.4	0.1

**Table 2:** Accuracy rate.

Accuracy		
Rounds	Random forest	SSR
0	0	0
200	0.2	0.4
400	0.4	0.7
600	0.5	1
800	0.6	1
1000	0.7	1

**Table 3:** True positive rate.

True positive rate		
Rounds	Random forest	SSR
0	0	0
200	0.1	0.3
400	0.2	0.6
600	0.3	0.9
800	0.4	1
1000	0.4	1

**Table 4:** Precision rate.

Precision	
Rounds	SSR
0	0
200	0.2
400	0.3
600	0.7
800	0.9
1000	1

**Table 5:** Recall.

Recall value	
Rounds	SSR
0	0
200	0.2
400	0.4
600	0.58
800	0.9
1000	1

## Data Availability

The data used to support the study will be made available from the corresponding author upon reasonable request.
